# Risk factors for excessively prolonged meropenem use in the intensive care setting: a case-contol study

**DOI:** 10.1186/s12879-017-2229-8

**Published:** 2017-02-08

**Authors:** Juri Katchanov, Benno Kreuels, Florian P. Maurer, Kai Wöstmann, Johannes Jochum, Christina König, Kariem Seoudy, Holger Rohde, Ansgar W. Lohse, Dominic Wichmann, Michael Baehr, Camilla Rothe, Stefan Kluge

**Affiliations:** 1 Department of Intensive Care Medicine, University Medical Center Hamburg-Eppendorf, Martinistr. 52, 20246 Hamburg, Germany; 2 Division of Infectious Diseases and Tropical Medicine, First Medical Department, University Medical Center Hamburg-Eppendorf, Hamburg, Germany; 3 Department of Medical Microbiology, Virology and Hygiene, University Medical Center Hamburg-Eppendorf, Hamburg, Germany; 4 Hospital Pharmacy, University Medical Center Hamburg-Eppendorf, Hamburg, Germany; 5 Infectious Disease Epidemiology, Bernhard-Nocht-Institute for Tropical Medicine, Hamburg, Germany

**Keywords:** Antimicrobial use, Antimicrobial stewardship, Broad-spectrum antibiotics

## Abstract

**Background:**

Inappropriate use of broad-spectrum antimicrobials affects adversely both the individual patient and the general public. The aim of the study was to identify patients at risk for excessively prolonged carbapenem treatment in the ICU as a target for antimicrobial stewardship interventions.

**Methods:**

Case–control study in a network of 11 ICUs of a university hospital. Patients with uninterrupted meropenem therapy (MT) > 4 weeks were compared to controls. Controls were defined as patients who stayed on the ICU > 4 weeks and received meropenem for ≤ 2 weeks. Associations between case–control status and potential risk factors were determined in a multivariate logistic regression model.

**Results:**

Between 1^st^ of January 2013 and 31^st^ of December 2015, we identified 36 patients with uninterrupted MT > 4 weeks. Patients with prolonged MT were more likely to be surgical patients (72.2% of cases vs. 31.5% of controls; p ≤ 0.001) with peritonitis being the most common infection (n = 16, 44.4%). In the multivariate logistic regression model colonization with multidrug-resistant (MDR) Gram-negative bacteria (OR 7.52; 95% CI 1.88–30.14, p = 0.004) and the type of infection (peritonitis vs. pneumonia: OR 16.96, 95% CI 2.95–97.49) were associated with prolonged MT.

**Conclusion:**

Surgical patients with peritonitis and patients with known colonization with MDR Gram-negative bacteria are at risk for excessively prolonged carbapenem therapy and represent an important target population for antimicrobial stewardship interventions.

## Background

Antibiotics are among the most frequently prescribed drugs in intensive care settings [1]. Administration of antibiotics in patients has been shown to be an important risk factor for the emergence of colonization and infection with antibiotic-resistant bacteria [1–4]. Furthermore, prolonged use of antimicrobials is associated with *Clostridium difficile* infection, antibiotic-related adverse events, and increased health care costs [2]. Prolonged use of broad-spectrum antibiotics such as carbapenems is particularly worrying as it promotes the spread of multidrug-resistant, difficult-to-treat pathogens.

In the present study, we analysed retrospectively patients treated with meropenem in a large centre for intensive care medicine in a German university hospital. We sought to describe the characteristics of patients having received excessively prolonged meropenem treatment, defined as uninterrupted therapy > 4 weeks, their microbiological data, and outcome on the ICU. We chose the duration of > 4 weeks as it indicates an inappropriately long use of a broad-spectrum antimicrobial not supported by any guideline or local standard procedure. Our aims were i) to understand risk factors for excessively prolonged treatment with a carbapenem and ii) to identify target populations for pre-emptive antimicrobial stewardship interventions.

## Methods

The University Medical Center Hamburg-Eppendorf is a tertiary level medical centre with approx. 1600 hospital beds and 80,000 admissions per year. The Department of Intensive Care includes 11 ICUs with a capacity of 132 beds. Approximately 8,000 patients are admitted to the ICUs each year, with an average length of stay on the ICU of 4.5 days. All decisions to initiate or to discontinue antimicrobial treatment are taken by the ICU consultants.

Meropenem was the only hospital-listed standard carbapenem during the study period (1^st^ of January 2013 until 31^st^ of December 2015). In selected cases, imipenem/cilastin was used on special request. These cases were excluded from further analysis, as they did not represent the standard prescription policy and made up less than 7% of all carbapenem prescriptions. Doripenem and ertapenem were not used during the study period.

### Patient identification from database

Basic data on all ICU patients who received meropenem during the study period were retrieved from the electronic medical records (ICM, Dräger, Lübeck, Germany). Patients were grouped according to the length of ICU stay (≤1 day, > 1 to 3 days, > 3 to 7 days, > 7 to 14 days, > 14 to 28 days, and > 28 days). The following information was additionally retrieved for patients who remained in the ICU for longer than 4 weeks: age (grouped as <50, 50–70 and >70 for regression analysis), sex, main diagnosis defined as the main reason for ICU admission (grouped as medical, surgical, neurosurgical-neurological), presence of malignancy, Simplified Acute Physiology Score (SAPS score, grouped as below median (<40) and above median (≥40) for regression analysis), antibiotic treatment, surgical procedures including re-operation to obtain source control, results of source control management and outcome in the ICU.

### Case–control design

Cases were defined as patients who stayed on the ICU longer than 4 weeks and received meropenem for longer than 4 weeks without interruption. If a patient was on meropenem therapy while being transferred to the ICU, the duration of meropenem therapy before admission was added to the duration of therapy during the ICU stay.

Controls were defined as patients who stayed on the ICU longer than 4 weeks and were treated with meropenem for 2 weeks or less. Meropenem therapy (MT) for ≤ 2 weeks was used as a marker for guideline-adherent prescription for ventilator-associated pneumonia, urinary tract infection and peritonitis [5–8]. To attain optimal statistical power, 108 controls were randomly selected from 322 patients fulfilling the definition (3:1 ratio of controls to cases) by use of a random number generator.

### Data on antimicrobial resistance

All available microbiological data generated during the hospital stay were collected for patients included in the study. In addition, all patients were screened on admission to the ICU for colonization with the following bacteria: nasal swab for methicillin-resistant *S. aureus* (MRSA), wound swabs for MRSA, pharyngeal and deep anal (anorectal) swabs for vancomycin-resistant enterococci (VRE) and multidrug-resistant (MDR) Gram-negative bacteria. MDR Gram-negative bacteria (MRGN) were defined as 3MRGN or 4MRGN in accordance to the current definition by the German national public health institute (Robert Koch Institute). In brief, Enterobacteriaceae are defined as 3MRGN when resistant to ureidopenicillin/beta-lactamase inhibitor combinations (e.g. piperacillin/tazobactam), third generation cephalosporins (e.g. ceftriaxone) and fluoroquinolones (e.g. ciprofloxacin), but susceptible to carbapenems (e.g. meropenem). 4MRGN Enterobacteriaceae are defined as being resistant to ureidopenicillins/beta-lactamase inhibitor combinations, third generation cephalosporins, fluoroquinolones and carbapenems.

### Statistical analysis

Summary statistics for the study participants were expressed as proportions for dichotomous or categorical variables with a corresponding Chi^2^ test and as medians with interquartile range (IQR) for continuous variables with a corresponding Wilcoxon rank-sum test.

Possible associations between case–control status and potential risk factors were determined by calculating odds ratios (OR) and 95% confidence intervals (CI). In a first step, analysis was performed as univariate logistic regression with the case–control status as independent variable and potential risk factors as dependent variables (i.e. age, sex, main reason for ICU admission, presence of malignancy, SAPS score, colonization with carbapenem-susceptible, MDR bacteria, type of infection). All variables showing at least some evidence of an association with case–control status (defined as p < 0.1) were then included in a multivariate logistic regression model to adjust for potential confounding. All data analyses were performed with Stata 12 (StataCorp LP, College Station, USA).

## Results

### Selection of cases and controls

**Fig. 1:**
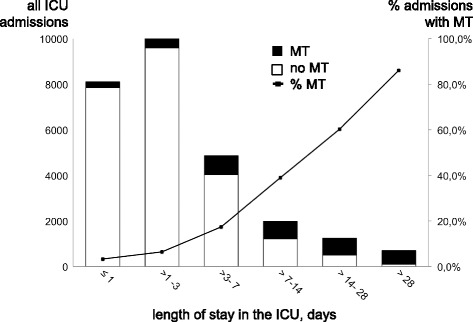
Usage of meropenem in relation to the length of stay (LOS) on the ICU. The bar chart depicts all ICU admissions during 2013–2015 (*left vertical axis*), *black bars* indicate the number of admissions with meropenem therapy (MT). The line graph depicts the percentage of admissions treated with meropenem (*right vertical axis*) in each LOS group

**Fig. 2:**
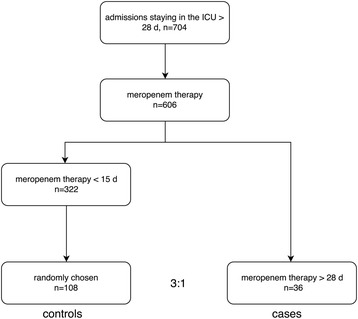
Patients disposition

In total, 3,909 patients admitted to the ICUs during the study period were treated with meropenem. The proportion of meropenem prescriptions increased with increasing length of stay (LOS), ranging from 6.4% in patients staying between 24 and 72 h to 86.1% (606/704 patients) in patients with an ICU LOS of longer than 4 weeks (Fig. 1). 322 patients with ICU LOS > 4 weeks received uninterrupted MT ≤ 2 weeks, and 108 of these patients were randomly selected as controls (Fig. 2). 36 patients received uninterrupted MT for longer than 4 weeks. One patient received meropenem twice during admission and the longer episode was counted.

### Characteristics of cases and controls

The characteristics of cases and controls are summarized in Table 1. There were no differences in age, sex, and simplified acute physiologic score (SAPS) at admission. Cases were more likely to be surgical patients (72.2% of cases vs. 31.5% of controls; p ≤ 0.001), while controls were more likely to be patients with medical conditions (11.1% of cases vs. 42.7% of controls; p = 0.001). None of the surgical patients with peritonitis was considered to have the source of the abdominal infection adequately controlled. This fact is reflected by the very high number of re-operations in this group. Cases suffered more often then controls from a malignant disease (47.2% of cases vs. 20.4% of controls; p = 0.002). All malignancies in the prolonged MT were solid tumours (n = 17), whereas in controls 4 patients had a haematological malignancy and 18 patients a solid tumour.

**Table 1:** Clinical characteristics of patients with prolonged meropenem therapy (MT) > 4 weeks and controls (ICU-stay > 4 weeks and meropenem therapy ≤ 14 days)

	Cases^a^	Controls^b^	*p*
Number of patients	36	108	
Duration of uninterrupted meropenem use, days, median (IQR)	34 (31–39)	9 (7–11)	<0.001
Age in years, median (IQR)	63 (51–71)	66 (56–73)	0.26
Female sex, *n* (%)	13 (36.1)	36 (33.3)	0.76
Days on ICU, median (IQR)	66 (51.5–92.5)	35 (31–45)	<0.001
Malignancy, *n* (% of all pts)	17 (47.2)	22 (20.4)	0.002
SAPS score at admission, median (IQR)	42 (35–48)	39 (33–47)	0.36
Medical patients, *n* (% of all pts)	4 (11.1)	45 (42.7)	0.001
Neurological patients, *n* (% of all pts)	6 (16.7)	30 (26.9)	0.22
Surgical patients, *n* (% of all pts)	26 (72.2)	34 (31.5)	<0.001
Number of operations, median (IQR)^c^	14 (5–27)	4 (3–7)	<0.001
Colonization/infection with carbapenem-susceptible, multidrug-resistant Enterobacteriaceae *n* (%)	7 (19.4)	7 (6.5)	0.007
Type of infection, *n* (%)			
pneumonia	4 (11.1)	27 (25.0)	<0.001
peritonitis	16 (44.4)	5 (4.6)	
mediastinitis	6 (16.7)	4 (3.7)	
unclear focus	10 (27.8)	72 (66.7)	
In-ICU-mortality, n (%)	16 (44.4%)	27 (25.0%)	0.03

^a^patients with ICU stay > 4 weeks and MT > 28 days

^b^patients with ICU stay > 4 weeks and MT ≤ 14 days

^c^in surgical patients

Abbreviations: *IQR*: interquartile range *SAPS*: simplified acute physiology score *MT*: meropenem therapy

Cases were colonized with 3MRGN bacteria more often than controls (p = 0.007) and had a higher mortality on ICU (p = 0.03).

### Risk factors for prolonged MT

Univariate logistic regression showed an association between prolonged MT and the presence of malignancy (OR 3.5; 95% CI 1.57-7.82, p = 0.002), colonization with multidrug-resistant carbapenem-susceptible Gram-negative bacteria (OR 4.12; 95% CI 1.38-12.35, p = 0.01), being a surgical patient as compared to being a medical patient (OR 8.36, 95% CI 2.67-26.17, p < 0.001) and the type of infection (p < 0.001) with a strongly increased risk for patients with peritonitis compared to patients with pneumonia (OR 21.60, 95% CI 5.05-92.35). No association was seen for age (p = 0.44), sex (p = 0.76) and SAPS score (p = 0.33) at admission. In the multivariate logistic regression model colonization with carbapenem-susceptible, multidrug-resistant Enterobacteriaceae (OR 7.52; 95% CI 1.88-30.14, p = 0.004) and the type of infection (peritonitis vs. pneumonia: OR 16.96, 95% CI 2.95-97.49) were associated with prolonged MT (Table 2).

**Table 2:** Analysis of potential risk factors for excessively prolonged meropenem therapy

		*Univariate logistic regression*			*Multivariate logistic regression*		
		*OR*	*95* % *CI*	*P*-*value*	*OR*	*95% CI*	*P-value*
age	<50	1		0.44	-	-	-
	50-70	0.72	0.25–2.04				
	≥70	0.49	0.16–1.53				
sex	male	1		0.76	-	-	-
	female	1.13	0.51–2.49				
malignancy	No	1		0.002	1		0.84
	Yes	3.5	1.57–7.82		1.14	0.32–4.06	
SAPS at admission	<40	1		0.33	-	-	-
	≥40	1.45	0.68–3.11				
patient group	medical	1		<0.001	1		0.31
	neurological	2.41	0.63–9.30		2.79	0.65 – 11.92	
	surgical	8.36	2.67–26.17		2.46	0.50 – 11.98	
colonization with carbapenem-susceptible, MDR bacteria	no	1		0.01	1		0.004
	yes	4.12	1.38–12.35		7.52	1.88–30.14	
type of infection	pneumonia	1		<0.001	1		<0.001
	peritonitis	21.60	5.05–92.35		16.96	2.95 – 97.49	
	mediastinitis	10.12	1.96–52.41		6.27	0.81 – 48.76	
	unclear focus	0.94	0.27–3.24		0.77	0.21 – 2.84	

Full model containing all variables with at least some evidence of association (*p* < 0.1) with the outcome in univariate regression analysis

Abbreviations: *MDR* multidrug-resistant; *OR*: odds ratio; *95% CI*: 95% confidence Interval

### Microbiological data

In the prolonged MT group, 6 patients were colonized with 3MRGN *E. coli* and 1 patient with 3MRGN *K. pneumonia*. In the control group, 4 patients were colonized with 3MRGN *E. coli*, 2 patients with 3MRGN *K. pneumoniae* and 1 patient with 3MRGN *K. oxytoca*. One patient in the prolonged MT group colonized with 3MRGN *E. coli* and carbapenem-susceptible *Enterobacter cloacae* developed colonization with carbapenem-resistant *E.coli* and *E. cloacae* during prolonged MT. In one patient from the prolonged MT group, emergence of carbapenem-resistant *P. aeruginosa* was detected during MT (anal swab).

Bacterial isolates detected in intraoperative specimens, blood and cerebrospinal fluid (CSF) cultures of patients with prolonged MT are summarized in Table 3.

**Table 3:** Positive bacterial culture results from sterile sites at onset and during the course of prolonged meropenem therapy

Intraoperative cultures (abdominal, thoracic)	blood cultures	CSF, brain tissue
*Enterococcus faecium*, n = 7 *Enterococcus faecalis*, *n* = 6 *Stenotrophomonas maltophilia*, n = 6 multidrug-resistant, carbapenem-susceptible *Escherichia coli*, n = 2^a^ *Enterobacter cloacae complex*, *n* = 2 Coagulase-negative staphylococci, *n* = 2 *Pseudomonas aeruginosa*, *n* = 1 *Proteus mirabilis*, *n* = 1 *Proteus vulgaris*, *n* = 1 *Klebsiella pneumoniae*, *n* = 1 *Escherichia coli (non MRGN)*, *n* = 1 *Staphylococcus aureus*, *n* = 1 *Enterococcus gallinarum*, *n* = 1	*E. faecalis*, n = 1 *E. faecium*, *n* = 1 Vancomycin-resistant *Enterococcus faecium*, *n* = 1 *E. aerogenes*, *n* = 1 *E. cloacae*, *n* = 1 *S. maltophilia*, *n* = 1 multidrug-resistant, carbapenem-resistant *P.aeruginosa*, *n* = 1	*Staphylococcus aureus*, n = 1 *Staphylococcus epidermidis*, *n* = 1 *Proteus mirabilis*, *n* = 1 *Bacteroides fragilis*, *n* = 1

^a^isolates that require unequivocally carbapenem therapy

Abbreviations: *n* number of patients; *CSF* cerebrospinal fluid

## Discussion

In our study, surgical patients with intra-abdominal infections were at risk for excessively prolonged meropenem use. The majority of the patients presented with an anastomotic leak requiring repetitive surgical interventions for source control. Around half of the patients had an initial surgical procedure for gastrointestinal malignancy.

The duration of antimicrobial therapy in complicated intra-abdominal infection has been recently studied in a large randomized controlled trial [9]. In patients with an adequate source control procedure, the outcomes after fixed-duration antibiotic therapy of 4 days did not differ from those after a longer course of antibiotics of 8 days [9]. In our patients, repetitive surgery reflected difficulties in source control and the presence of persistent peritonitis. The duration of antibiotic therapy for persistent peritonitis is not well established [10], the recommendation in Germany being 7 to 10 days [8]. A longer duration of antibiotic therapy for intra-abdominal infections is an independent risk factor for subsequent intra-abdominal infections and associated with increased mortality [11].

According to the guidelines of Infectious Diseases Society of America (IDSA) empiric antibiotic therapy for health care–associated intra-abdominal infection should be driven by local microbiology results [12]. In the era of multidrug-resistance, empirical coverage for MDR bacteria may be prudent [10, 13–15]. In our group of surgical patients, MDR carbapenem-susceptible (so-called “3MRGN”) isolates were found in 6 patients (23.1%). All of them were known to be colonized with 3MRGN *Enterobacteriaceae* before or at onset of MT. However, only in 2 of these 6 patients 3MRGN *E. coli* was also detected in intraoperative swabs. In consequence, known colonization - and not necessarily infection - with 3MRGN *Enterobacteriaceae* was a risk factor for prolonged MT in our study. These findings are in accordance with studies that show that intensivists are more reluctant to de-escalate antimicrobial therapy in patients with known MDR colonization [16]. Retrospectively, the number of patients with relevant bacterial isolates at the site of infection and a clear indication for meropenem treatment was low (Table 3).

The in-ICU-mortality in patients with excessively prolonged MT was double as high as in the control group. It is likely that severe clinical condition prevented the intensivists from discontinuing meropenem. Of note, presence of systemic illness in complicated intra-abdominal infections does not necessitate a longer course of antimicrobial therapy if source control is obtained [17]. The majority of surgical patients with excessively prolonged MT had numerous re-operations reflecting difficulties in source control. In fact, in all patients with peritonitis and excessively prolonged MT the source control was considered as inadequate by the intensivists in charge. Inability to obtain source control is one of the main determinants of poor outcome [15]. However, prolonged broad-spectrum antimicrobial therapy is not likely to contribute to source control [18, 19]. There is a growing body of evidence that prolonged antimicrobial therapy is not beneficial for critically ill patients (reviewed in [20]). This might be particularly true for broad-spectrum antimicrobials where disadvantages of antibiotic therapy might outweigh unlikely benefits for the patient [21].

Our study has several limitations. It is a single centre study in a tertiary care hospital and our experience might not be representative for all ICU departments. The retrospective nature of the study does not allow assessment of the contribution of extensively prolonged MT to clinical outcome. Based on our definition of cases and controls, the data can be used to identify risk factors for excessively prolonged use of meropenem (>28 days) compared to guideline adherent use (≤14 days). However, we cannot draw conclusions on risk factors for slightly longer use than recommended by guidelines (i.e. 14–28 days). This group of patients is surely more heterogeneous than the cases in this study and should be included in the focus of future, larger studies.

Due to the small number of patients the power of the study was low, as reflected by wide 95% confidence intervals and several potential risk factors may not have been identified. Due to the low number of patients it was not possible to study the emergence of carbapenem-resistant (so-called 4MRGN) microorganisms under prolonged MT. However, detection of carbapenem-resistant *E. coli* and *E. cloacae* isolates after prolonged MT in one patient with known colonization with multidrug-resistant carbapenem-susceptible isolates and the emergence of carbapenem-resistant *P. aeruginosa* in another patient are worrying signs [22, 23].

## Conclusion

Surgical patients with persistent peritonitis are at risk for excessively prolonged carbapenem therapy. Antimicrobial stewardship programmes should target this patient group to prevent overuse of last resort antimicrobials.

## Abbreviations

CSF: Cerebrospinal fluid
ICU: Intensive care unit
IDSA: Infectious Diseases Society of America
LOS: Length of stay
MDR: Multidrug-resistant
MRGN: Multidrug-resistant Gram-negative bacteria
MRSA: Methicillin-resistant *S. aureus*
MT: Meropenem therapy
OR: Odds ratio
SAPS: Simplified Acute Physiology Score
VRE: vancomycin-resistant enterococci
